# Connectivity‐based segmentation of the periaqueductal gray matter in human with brainstem optimized diffusion MRI

**DOI:** 10.1002/hbm.22855

**Published:** 2015-07-02

**Authors:** Martyn Ezra, Olivia Kate Faull, Saad Jbabdi, Kyle Thomas Pattinson

**Affiliations:** ^1^ Nuffield Department of Clinical Neurosciences, Oxford Centre for Functional Magnetic Resonance Imaging of the Brain University of Oxford Oxford, Oxfordshire United Kingdom

**Keywords:** diffusion, magnetic resonance imaging, periaqueductal gray, pain, cardiovascular, segmentation, brainstem, human, respiratory, fear

## Abstract

The periaqueductal gray matter (PAG) is a midbrain structure, involved in key homeostatic neurobiological functions, such as pain modulation and cardiorespiratory control. Animal research has identified four subdivisional columns that differ in both connectivity and function. Until now these findings have not been replicated in humans. This study used high‐resolution brainstem optimized diffusion magnetic resonance imaging and probabilistic tractography to segment the human PAG into four subdivisions, based on voxel connectivity profiles. We identified four distinct subdivisions demonstrating high spatial concordance with the columns of the animal model. The resolution of these subdivisions for individual subjects permitted detailed examination of their structural connectivity without the requirement of an a priori starting location. Interestingly patterns of forebrain connectivity appear to be different to those found in nonhuman studies, whereas midbrain and hindbrain connectivity appears to be maintained. Although there are similarities in the columnar structure of the PAG subdivisions between humans and nonhuman animals, there appears to be different patterns of cortical connectivity. This suggests that the functional organization of the PAG may be different between species, and as a consequence, functional studies in nonhumans may not be directly translatable to humans. This highlights the need for focused functional studies in humans. *Hum Brain Mapp 36:3459–3471, 2015*. © **2015 The Authors Human Brain Mapping Published by Wiley Periodicals, Inc**.

## INTRODUCTION

The periaqueductal gray matter (PAG) is a poorly differentiated midbrain structure, known to be involved in a number of key homeostatic neurobiological functions, such as pain modulation and cardiorespiratory control [Linnman et al., 2012]. Situated at the confluence of ascending sensory and descending higher center pathways, the PAG plays an essential role in the integrations of these inputs [Benarroch, 2012].

**Figure 1:**
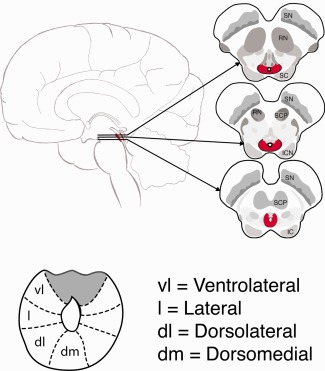
a. Position of PAG within the midbrain surrounding the cerebral aqueduct. **b**. Divisions of the PAG derived from animal models. [Color figure can be viewed in the online issue, which is available at http://wileyonlinelibrary.com.]

Currently, most of our understanding of its structure and function comes from animal research. Despite its cytoarchitectonic homogeneity, animal models have shown significant heterogeneity with respect to anatomical connections, functional, and chemical properties between subdivisions of the PAG [Dampney et al., 2013]. These subdivisions are proposed as four longitudinal columns parallel to the aqueduct: the dorsomedial (dmPAG), dorsolateral (dlPAG), lateral (lPAG), and ventrolateral (vlPAG) (Fig. 1). The ventromedial aspect is not considered part of the PAG and is comprised of discrete nuclei.

The ability to differentiate these distinct subdivisions in vivo is critical to improving our understanding of the PAG and the efficacy of interventional therapies such as chronic pain modulation using deep brain stimulation (DBS). Thus, far attempts to segment the human PAG have yielded disappointing results in part due to its small size and location within the brainstem surrounding the cerebral aqueduct. Structural magnetic resonance imaging (MRI) studies have failed to differentiate subdivisions even in high‐resolution ex vivo studies [Lambert et al., 2013]. Functional imaging techniques [positron emission tomography (PET), functional magnetic resonance imaging (fMRI)] do not yet possess the resolution required [Linnman et al., 2012], althought 7T fMRI is beginning to show promise [Faull et al., 2015].

Tracer studies performed in animals have demonstrated that subdivisions of the PAG have different anatomical connection patterns. This suggests the possibility of segmenting the human PAG based on anatomical connections to other areas of the brain. Diffusion‐based tractography is a noninvasive MRI technique able to identify inter‐regional white matter connectivity in vivo and has been successfully applied in several studies to segment other subcortical regions such as the thalamus [Johansen‐Berg et al., 2005] and substantia nigra [Menke et al., 2010].

There have been a limited number of tractography studies of the human PAG, predominantly examining gross PAG cortical and subcortical connectivity [Linnman et al., 2012]. Only one study [Pereira et al., 2010] has investigated heterogeneity of anatomical connectivity within the PAG. However, this study was limited by only examining the ventral and dorsal aspects of the PAG and in the a priori selection of the seed locations. Interestingly they identified difference in connectivity, which did not completely match the nonhuman tracer studies.

It is the aim of this study to utilize high‐resolution, brainstem optimized diffusion MRI to classify PAG voxels according to their connectivity profiles, and thus, segment the PAG into the four distinct connectivity defined regions predicted by the animal model of the PAG. These derived regions will be formed without a priori knowledge of their locations or structure and will enable detailed examination of the connectivity properties of the human PAG. We hypothesized that the connectivity defined regions, would correspond in spatial location to the animal model. However, these regions may demonstrate different connectivity profiles based on human variation observed in previous tractography studies.

## MATERIALS AND METHODS

### Subjects

Nineteen healthy subjects were included in this study (6 women and 13 men; mean ± SD age, 31.1 ± 5.2 years; range, 23–40 years; all right handed). All volunteers were screened for MR compatibility and scanned with ethical approval and informed consent in accordance with the Oxfordshire Clinical Research Ethics Committee. The research materials supporting this publication can be accessed by contacting martyn.ezra@conted.ox.ac.uk.

### Image Acquisition

All images were acquired on a Siemens (Erlangen, Germany) Trio 3T scanner with a 12‐channel head coil. Diffusion weighted images were acquired in the axial plane using an echo planar imaging sequence (3 acquisitions of 60 directions with 5 nondiffusion weighted images, *b*‐value 1,000 s mm^−2^, voxel size 1.5 × 1.5 × 1.5 mm, 100 slices). Field of view incorporated the whole brain including brainstem. Cardiac gating was performed to minimize artifacts from pulsatile flow of the cerebrospinal fluid of which the brainstem is particularly sensitive [Brooks et al., 2013; Harvey et al., 2008]. Brainstem optimization has resulted in a unique high quality dataset that is not available on public databases. Each subject also had a T1 weighted high‐resolution (1 × 1 × 1 mm voxels) structural image acquired to aid registration.

### Diffusion MRI Data Preprocessing

Preprocessing was performed using FMRIB's diffusion toolbox (FDT) in the FSL software package (http://www.fmrib.ox.ac.uk/fsl/). This included extraction of non‐brain tissue using brain extraction tool (BET) [Smith, 2002] and affine registration to a reference volume to correct for eddy currents and head motion using EDDYCORRECT [Jenkinson and Smith, 2001]. The data from the three acquisitions for each subject were averaged to improve the signal to noise ratio and voxelwise estimates of fiber orientation and uncertainty was carried out using BEDPOSTX [Behrens et al., 2003b, 2007].

### Image Registration

After preprocessing, each subject's diffusion weighted scans were registered to the MNI152 1 mm standard space (average T1 brain image constructed from 152 normal subjects at the Montreal Neurological institute, Montreal, QC, Canada). Registration was performed as a three‐step procedure via the high‐resolution T1‐weighted structural image with linear registration using FLIRT and nonlinear registration using FNIRT [Jenkinson and Smith, 2001; Jenkinson et al., 2002].

### Definition of Seed and Target Masks

The PAG seed mask was drawn by hand using FSLview in FSL (http://www.fmrib.ox.ac.uk/fsl/). Each subject had a left and right PAG mask drawn in diffusion space using the B0 image as a template; this was made with reference to Duvernoy's atlas of the Human Brainstem and Cerebellum [Duvernoy, 2009]. The mask represents a conservative estimate of the PAG, to reduce contamination of our results by inclusion of adjoining areas.

The cortical and subcortical masks were defined from the Harvard Oxford cortical and subcortical structural atlases (part of FSLview), which are population‐based probability atlas in MNI152 standard space. Masks were thresholded to include only voxels estimated at greater than 50% of probability of being in that structure. Masks of the medulla, pons and PAG were drawn in MNI152 standard space using FSLview with reference to Duvernoy's atlas of the Human Brainstem and Cerebellum [Duvernoy, 2009]. The hypothalamus mask was drawn in reference to an MRI atlas of the hypothalamus [Baroncini et al., 2012].

### Probabilistic Tractography

Probabilistic tractography was carried out for each subject using previously described methods [Behrens et al., 2003b, 2007] with FDT (http://www.fmrib.ox.ac.uk/fsl/) with 10,000 samples per voxel. Estimates of the connections between each voxel in the PAG seed region and every voxel of the whole brain were then calculated. This generates a connectivity profile for each seed voxel and is derived from the number of samples that arrive at each target voxel. To reduce the false‐positive connections, the path distribution estimates were thresholded to a connection probability of *P* < 0.0003. A high sample number and low threshold was chosen to improve identification of small cortical tracts. A cross‐correlation matrix between the connectivity profiles of all voxels in the seed mask was then calculated [Johansen‐Berg et al., 2004].

### Tractography‐Based Segmentation

The cross‐correlation matrix was fed into a *k* means clustering algorithm [MacQueen, 1967]. *K* means treats each observation as having a location in space and uses an iterative algorithm to find partitions in which objects within each cluster are as close to each other, and as far from objects in other clusters as possible. The result is to cluster voxels together that share connectivity profiles.

*K* means clustering requires the number of clusters to be selected a priori, the animal model of the PAG has four distinct columns exist either side of the aqueduct. The left and right PAG were examined individually, with four clusters for each side being selected. Mapping the individual subject clusters on to a PAG mask drawn in MNI152 standard space and adding them together created group probability maps. This was performed by using linear transformation matrices generated by the registration of the individually drawn PAG masks in diffusion space to the PAG mask drawn in MNI152 standard space using FLIRT. This method was chosen opposed to using the whole brain registrations as it resulted in better spatial alignment of the results. The group probability maps for each cluster were thresholded to include >30% of the population.

### Selection and Thresholding of the Clusters

The results of the clustering were examined to determine if it had been successful at an individual subject level. Successful clustering was defined as identification of a distinct cluster that had a spatial representation as predicted by the animal model of the PAG, that is, a cluster that was parallel to the aqueduct in the dorsomedial, dorsolateral, lateral, or ventrolateral aspect of the PAG. Previous tractography segmentation studies [Behrens et al., 2003a] have identified that results were reproducible in approximately 70% of subjects due to the performance of tractography. *K* means clustering is a hard clustering technique that will identify a predetermined number of clusters. Therefore, arbitrary segmentation may take place in some subjects where tractography has been unable to identify any differences. To improve assessment of the connectivity profiles of the different columns, selection was performed to remove failed segmentation. This is based on the assumption that the human PAG is in concordance with the animal model. The clusters that were in concordance with the animal model of the PAG were selected for thresholding.

The human PAG is approximately 14 mm long and 4–5 mm wide either side of the aqueduct. Due to the spatial constraints of diffusion MRI (1.5 mm isotropic voxels) there is likely to be significant overlap between PAG columns within individual voxels. It is, therefore, necessary to threshold out the PAG voxels that poorly belong to any cluster. This was achieved by deriving the silhouette value for each voxel; this represents a measure of how similar that voxel is to other voxels in its own cluster, when compared to voxels in other clusters. Values below 0.25 were chosen to signify that the voxels were poorly differentiated [Kaufman and Rousseeuw, 1990]. The thresholded clusters were used for the generation of connectivity profiles and spatial maps.

### Connectivity Profiles

To test if the connectivity profiles of the different columns of the human PAG resemble those derived from nonhuman tracer studies, we analyzed the probabilistic connections between each PAG column and predefined target regions (Table 1). The target regions examined have been shown to exhibit PAG connectivity in previous animal and human studies. Total PAG connectivity was first calculated for each subject to each target region, using probabilistic tractography (10,000 samples per voxel). To reduce the false‐positive connections, target regions with average connection probability of *P* < 0.0003 were removed from the cluster connectivity profile analysis. Probabilistic tractography was then used to calculate the probability of connection between each column and the target region that survived thresholding for each subject. These results were then used to create anatomical connectivity profiles for each column, using the average relative connectivity between each column within the PAG to the individual target regions. Statistical analysis of connection probabilities was performed using SPSS 21.0 (SPSS). Repeated‐measures MANOVA was performed to visualize the anatomical connection probabilities differences across the four columns.

**Table 1:** Cortical and subcortical target regions known to demonstrate PAG connectivity in Humans and/or Animals

Cortical	Subcortical
Frontal Pole	Amygdala
Middle Frontal Gyrus	Hypothalamus
Superior Frontal Gyrus	Thalamus
Anterior Division of the Cingulate Gyrus	Pons
Paracingulate gyrus	Medulla
Precentral Gyrus	
Postcentral Gyrus	
Insular Cortex	
Superior Parietal Lobule	
Occipital Pole	

### Whole Brain Connectivity

To better assess the spatial patterns of whole brain connectivity to each PAG cluster we back‐projected the results of the clustering onto the brain. This method has been described in detail previously [Menke et al., 2010], but in brief it involves identifying which column each voxel in the brain demonstrates the strongest connectivity to (thresholded at *P* < 0.0003). The result is a spatial map of the brain that demonstrates which locations are most likely to connect to each of the divisions of the PAG. Transforming the individual subject spatial maps into MNI152 space and adding them together created group probability maps. The group probability maps for each spatial map were thresholded to include > 30% of the population.

## RESULTS

### Clustering

**Figure 2:**
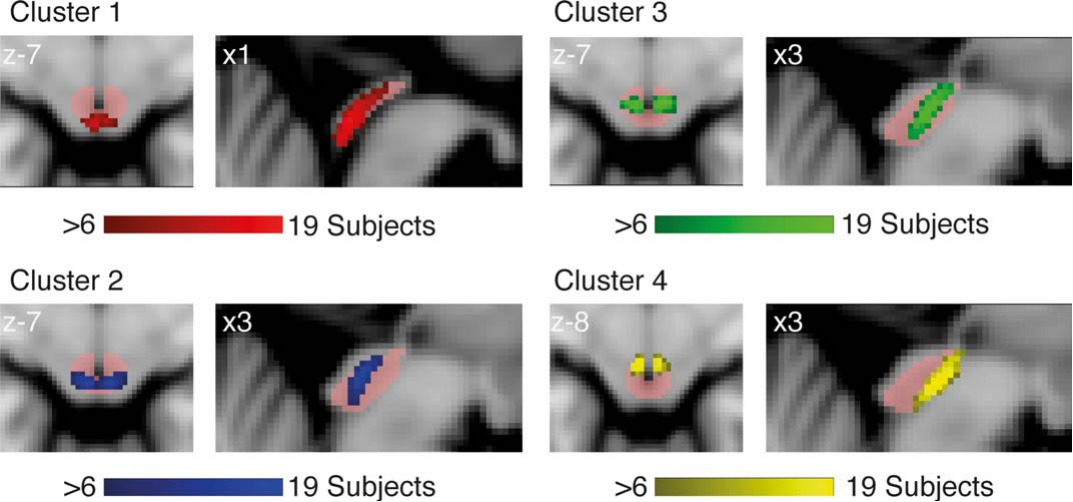
Group probability maps of clustering results over PAG mask (Pink). Axial and right sided sagittal (left side omitted as equivalent to right) slices taken at the average the center of gravity of the cluster(s). Coordinates given in anatomical space. Results thresholded to include >35% of the population. [Color figure can be viewed in the online issue, which is available at http://wileyonlinelibrary.com.]

**Figure 3:**
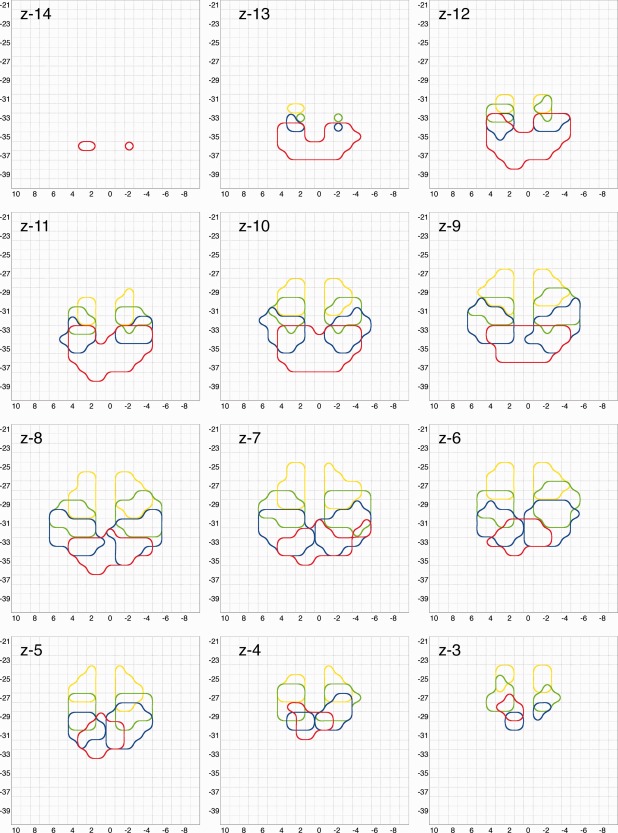
PAG connectivity atlas. Axial slices through the whole PAG showing edges of thresholded (>35% of the population) group probability maps. *X*‐ and *Y*‐axes give coordinates in anatomical space. *Z*‐coordinate of each slice is indicated in the top left corner. [Color figure can be viewed in the online issue, which is available at http://wileyonlinelibrary.com.]

Group probability maps of the clustering results for all subjects reproduced in MNI152 standard space demonstrated four distinct clusters either side of the cerebral aqueduct. These results demonstrate good spatial concordance with the columns derived from animal model of the PAG (Figs. 2 and 3). We suggest that cluster 1 (Red) is the dmPAG, cluster 2 (Blue) the dlPAG, cluster 3 (Green) the lPAG, and cluster 4 (Yellow) the vlPAG.

**Figure 4:**
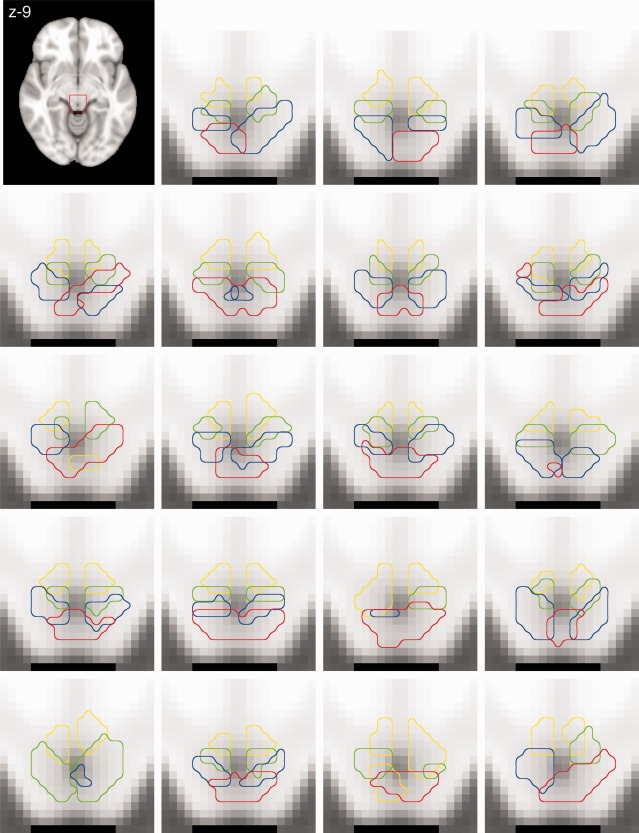
Connectivity‐based segmentation of the PAG in nineteen subjects. Top left panel indicates location of axial PAG slice with anatomical coordinate (taken at the midpoint of the PAG). Each subsequent panel represents data from an individual subject. [Color figure can be viewed in the online issue, which is available at http://wileyonlinelibrary.com.]

Performance of the tractography‐based *k*‐means segmentation at the subject level was assessed by visual inspection of the clusters in the subject's individual diffusion space. Successful clustering was defined as distinct clusters that have a spatial representation predicted by the animal model of the PAG. This was present in 100% of the left/right PAGs examined for cluster 4 (vlPAG). Clusters 1 (dmPAG), 2 (dlPAG), and 3 (lPAG) demonstrated successful clustering in 61%, 55%, and 84%, respectively (Fig. 4). The most common alternative spatial representation was a failure to segment cluster 1 (dmPAG) and cluster 2 (dlPAG) in parallel to the aqueduct but instead into rostral and cephalad clusters (26%).

### Connectivity Profiles

**Figure 5:**
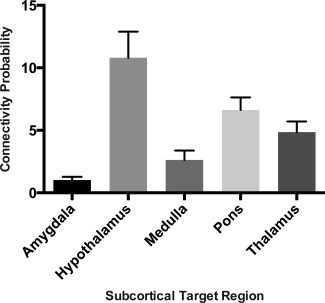
This graph displays the mean connectivity probability between the whole PAG seed region and the subcortical targets. Error bars demonstrate the standard error of the mean.

**Figure 6:**
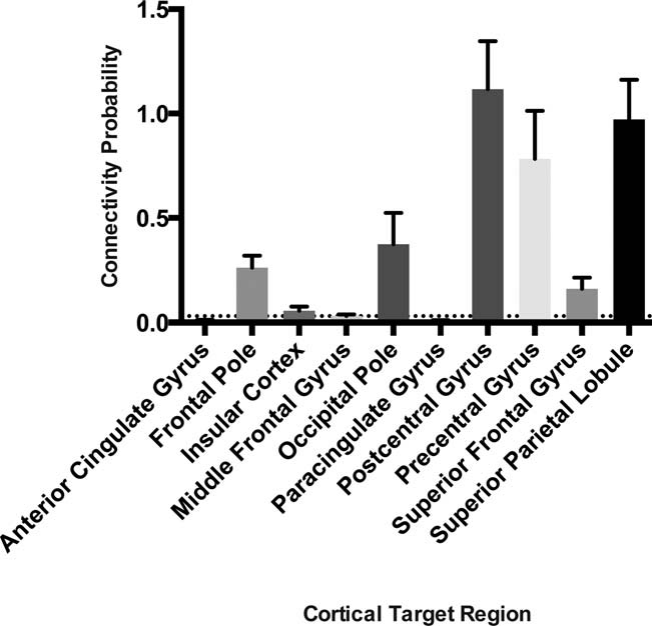
This graph displays the mean connectivity probability between the whole PAG seed region and the cortical targets and threshold level (dotted line). Error bars demonstrate the standard error of the mean.

The total PAG connectivity to the five subcortical (Fig. 5) and 10 cortical (Fig. 6) target regions demonstrates dominant connectivity of the PAG to the hypothalamus superiorly. The anterior division of the cingulate gyrus and paracingulate gyrus failed to reach the threshold of *P* ≥ 0.0003 and were removed from subsequent analysis.

**Figure 7:**
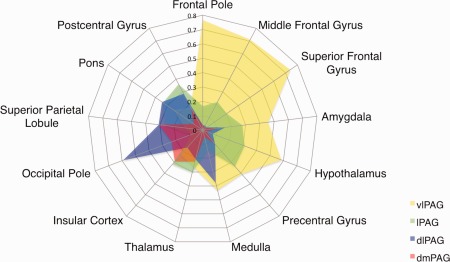
Radial diagram of relative connectivity of the clusters to predefined targets. [Color figure can be viewed in the online issue, which is available at http://wileyonlinelibrary.com.]

The relative connectivity of the columns to the target regions revealed different connectivity profiles for each cluster (Fig. 7). The vlPAG demonstrated dominant connectivity to the prefrontal cortical structures, hypothalamus, amygdala, precentral gyrus, and medulla. The lPAG had dominant postcentral gyrus and pontine connectivity, in addition to similar weaker connectivity to the prefrontal cortical areas, hypothalamus, and precentral gyrus. The dmPAG and dlPAG had similar patterns of cortical connectivity, but the dlPAG demonstrated distinct brainstem connectivity to the pons and medulla. The dlPAG also demonstrated stronger mean occipital pole connectivity; however, this was not statistically significant (Table 2), due to a large variance in occipital pole connectivity. Connectivity to the thalamus, and insular cortex was similar across all columns.

**Table 2:** Differences in anatomical connectivity probabilities for pairs of PAG columns, shown with *P* values (Bonferroni corrected)

	dmPAG vs. dlPAG	dmPAG vs. lPAG	dmPAG vs. vlPAG	dlPAG vs. lPAG	dlPAG vs. vlPAG	lPAG vs. vlPAG
Amygdala	0.63	0.291	0.001	0.017	<0.001	<0.001
Frontal Pole	0.685	0.002	0.001	0.002	0.001	<0.001
Hypothalamus	0.359	<0.001	<0.001	<0.001	<0.001	<0.001
Insular Cortex	0.247	0.418	0.54	0.45	0.801	0.318
Medulla	0.012	0.003	0.001	0.047	0.072	0.004
Middle Frontal	0.504	0.006	0.002	0.01	0.002	<0.001
Gyrus Occipital Pole	0.386	0.129	0.257	0.305	0.338	0.291
Pons	0.032	0.02	0.013	0.406	0.151	0.181
Postcentral Gyrus	0.139	0.18	0.547	0.974	0.003	<0.001
Precentral Gyrus	0.007	0.006	0.017	0.05	0.207	0.814
Superior Frontal Gyrus	0.426	0.01	0.023	0.009	0.028	0.009
Superior Parietal Lobule	0.783	0.582	0.066	0.069	0.001	0.005
Thalamus	0.589	0.362	0.622	0.079	0.417	0.447
Multivariate	0.124	0.031	<0.001	0.08	0.01	<0.001

Repeated measures MANOVA tests were performed to assess the differences between the columns with respect to connectivity probability to all the target regions. Univariate tests were also performed to test the difference between columns for each target region (Table 2). The results showed there was no significant difference between the dmPAG and dlPAG (*P* = 0.124) and also the dlPAG and lPAG (*P* = 0.08) when comparing the probability of connection to the predefined target regions. There was a significant difference between the dmPAG and lPAG (*P* = 0.031), dmPAG and vlPAG (*P* < 0.001), dlPAG and vlPAG (*P* = 0.01), and lPAG and vlPAG (*P* < 0.001).

### Back‐Projections

**Figure 8:**
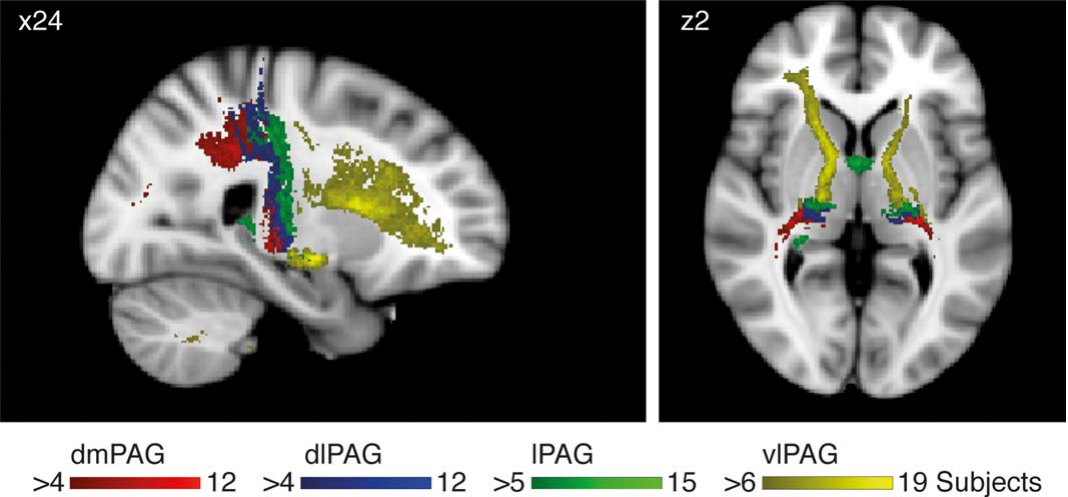
Axial and sagittal cortical slices in MNI152 standard space of group probability maps of back projections from all four PAG columns. Coordinates given in anatomical space. Results thresholded to include >35% of the population. [Color figure can be viewed in the online issue, which is available at http://wileyonlinelibrary.com.]

Whole brain back‐projections were used to assess the topographical distribution of the connectivity of each PAG column. Visualization of the cortical spatial maps revealed that the voxels most likely to connect to each PAG column had a similar distribution as identified by their connectivity profiles (Fig. 8).

Back‐projections were also used to examine the spatial patterns of connectivity of the different PAG columns within specific subcortical structures, known to have differential patterns of PAG connectivity.

#### Hypothalamus

**Figure 9:**
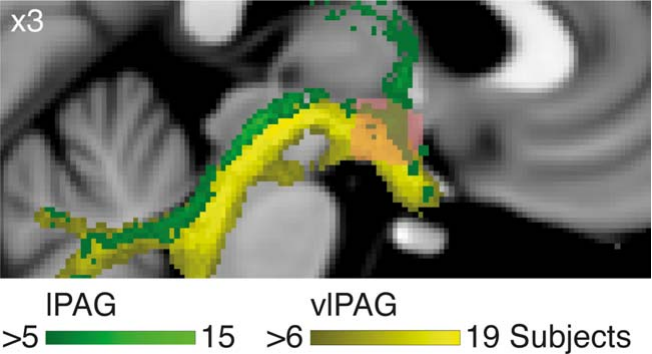
Sagittal slice of the hypothalamus in MNI152 standard space of group probability maps of back projections. lPAG (Green) passing through the dorsomedial hypothalamus and vlPAG (Yellow) passing through the ventromedial hypothalamus. Hypothalamus mask superimposed (Pink). Coordinates given in anatomical space. Results thresholded to include >35% of the population. [Color figure can be viewed in the online issue, which is available at http://wileyonlinelibrary.com.]

Within the hypothalamus vlPAG voxels were most likely to connect to voxels in the ventromedial hypothalamus, whereas lPAG voxels were most likely to connect to the dorsomedial hypothalamus (Fig. 9).

#### Midbrain

**Figure 10:**
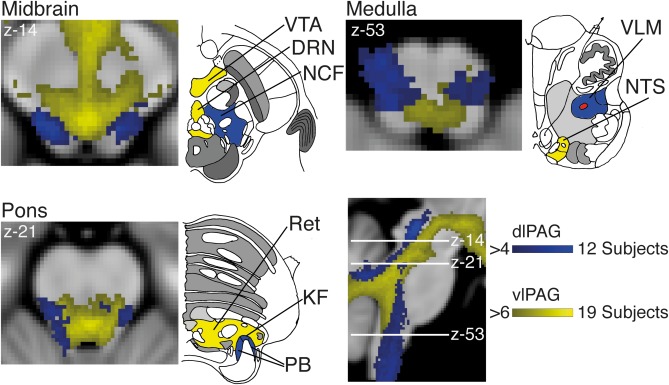
Axial slices in MNI152 standard space of group probability maps of back projections from dlPAG (Blue) and vlPAG (Yellow) columns. Results thresholded to include >35% of the population. Coordinates given in anatomical space. VTA, ventral tegmental area; DRN, dorsal nucleus raphe; NCF, nucleus cuneformis; Ret pontine reticular formation; KF, Kölliker‐Fuse; PB, parabrachial; VLM, ventrolateral medulla; NTS, nucleus tractus solitarius. Line drawing adapted from Duvernoy [Duvernoy, 2009]. [Color figure can be viewed in the online issue, which is available at http://wileyonlinelibrary.com.]

Within the midbrain, dlPAG voxels were most likely to connect to voxels of the nucleus cuneformis (NCF), where as the vlPAG voxels were most likely to connect to voxels of the ventral tegmental area (VTA) and the dorsal raphe nucleus (DRN) (Fig. 10).

#### Pons

In the dorsolateral pons, voxels were most likely to connect to the dlPAG. This region is consistent with locus ceruleus medially and parabrachial/Kölliker‐Fuse complex laterally. In the dorsomedial pons, voxels were most likely to the vlPAG. This region is consistent with the pontine reticular formation (Fig. 10).

#### Medulla

Within the medulla, the dorsolateral voxels containing the nucleus tractus solitarius, gracile nucleus, and dorsoreticular nucleus were most likely to connect to the vlPAG. The ventrolateral medulla (VLM) voxels were most likely to connect to the dlPAG (Fig. 10).

## DISCUSSION

High‐resolution brainstem optimized diffusion MRI has enabled the segmentation of the human PAG into four distinct subdivisions parallel to the cerebral aqueduct, similar to that identified in nonhuman studies. This has permitted detailed examination of their structural connectivity without requiring an a priori starting location.

### Clustering

At a group level, clustering was able to correctly identify four distinct clusters with a spatial representation predicted by the animal model of the PAG. At an individual subject level, clustering correctly identified these clusters in the majority of subjects. Although the clustering technique is unable to conclude how many columns the human PAG is derived from, while aiming to resolve four clusters, we identified a structure similar to that seen in the animal model.

Clustering was least successful in differentiating the dmPAG and dlPAG. The dmPAG and dlPAG possess similar patterns of cortical connectivity, but clear differences in brainstem connectivity. It is possible that the rostral‐cephalic segmentation observed in some subjects may not result from arbitrary incorrect segmentation but from the organization of inputs into the individual PAG columns. Animal studies have identified a rostral‐cephalic somatotrophic organization of inputs into the individual segments of the PAG, this may explain the pattern of segmentation observed. [Bandler et al., 2000; Keay and Bandler, 2001, 2002; Parry et al., 2008].

### Connectivity Profiles

Total PAG connectivity to the cortical and subcortical target regions was consistent with other diffusion MRI studies [Hadjipavlou et al., 2006; Owen et al., 2007, 2008; Pereira et al., 2010; Sillery et al., 2005]. Connectivity to the anterior division of the cingulate gyrus and paracingulate gyrus failed to reach the threshold, despite strong connectivity being demonstrated in animal tracer studies [An et al., 1998]. This has been previously noted and is thought to be due to the tracts perpendicular to, and passing through the corpus callosum bundle being blocked by large white matter tracts [Hadjipavlou et al., 2006].

Pereira et al. [2010], identified heterogeneity between dorsal and ventral PAG connectivity, which did not entirely correlate with animal studies. There are, however, limitations to their approach. Tractography was performed in healthy individuals, with seed locations derived from the mean electrode position of a different subject cohort receiving DBS. Differentiation of DBS electrode position used predefined anatomical relationships with other structures. This implies uniformity to the structure and position of the PAG, which cannot be assumed to be true. Therefore, the exact position of the seed location for tractography cannot be guaranteed. By segmenting the PAG using connectivity patterns and using these divisions to perform connectivity analysis we have overcome these limitations. We have been able to examine the connectivity of all four columns of the PAG without any prior assumptions of the location of different segments.

Patterns of cortical connectivity were generally consistent with the findings of animal tracer studies, with the exception of the prefrontal cortex (PFC). The precentral gyrus demonstrates dominant connectivity to the lPAG and vlPAG, and the occipital cortex strong connectivity to the dlPAG. This is in agreement with studies in rats; where primary motor areas project exclusively to the lPAG and vlPAG, while the secondary visual cortex preferentially innervate the dlPAG [Newman et al., 1989]. Furthermore, sensory cortex connectivity arose predominantly from the dlPAG and lPAG. This agrees with animal studies where dlPAG and lPAG receive somatotopically organized inputs from superficial nociceptors [Bandler et al., 2000; Keay and Bandler, 2001, 2002; Lumb, 2004; Parry et al., 2008].

Interestingly, we found significant differences in PFC connectivity. Tracer studies in macaques have shown distinct patterns of columnar PAG connectivity with different PFC structures. In animals, the dlPAG receives the dominant PFC input, primarily arising from the medial PFC. In contrast, the vlPAG receives input from orbital and anterior insular areas and the lPAG from the dorsomedial PFC [An et al., 1998]. Our results do not demonstrate the same columnar pattern of PFC connectivity, which arises predominantly from the vlPAG and partially from the lPAG, with minimal connectivity to the dlPAG.

Patterns dlPAG subcortical connectivity also differed to nonhuman studies. Hypothalamic and amygdala connectivity was modest when compared to the lPAG and vlPAG. This is in contrast to nonhuman studies in which some of the primary connections of the dlPAG arise from ventromedial hypothalamus via the amygdala [Motta et al., 2009]. Moreover, the spatial maps derived from the back‐projections, identified dominant vlPAG connectivity to the ventromedial hypothalamus, rather than the dlPAG [An et al., 1998]. Our findings identified dlPAG connectivity to the brainstem, previously not demonstrated in animals. However, this may result from connectivity via the NCF as diffusion MRI cannot determine if a tract is direct or indirect. Connectivity between the NCF and dlPAG, and between the NCF and brainstem has been demonstrated in animals [Bernard et al., 1989; Redgrave et al., 1988, 1990].

### Back‐Projections

The PAG is proposed to function by orchestrating different coping strategies when exposed to external stressors. Differential PAG column connectivity to nuclei within the brainstem and subcortical structures is thought to play a critical role in orchestrating these differing responses.

Human DBS and animal studies have identified that dlPAG activation triggers active coping strategies, involving sympathoexcitation, hyperventilation and short‐duration, non‐opioid‐mediated analgesia [Bandler et al., 2000; Green et al., 2005; Keay and Bandler, 2001; Pereira et al., 2010]. Our results are consistent with these observations. Within the midbrain dlPAG demonstrates connectivity with the NCF. This structure has nocifensive functions [Haws et al., 1989] and as previously discussed connectivity to the dlPAG. Furthermore, the dlPAG demonstrates connectivity to brainstem regions integral to cardiorespiratory control (either directly or indirectly). The dorsolateral pontine connectivity is likely to represent the locus ceruleus and parabrachial/Kölliker‐Fuse complex, nuclei responsible for sensory processing of respiratory signals [Smith et al., 2009]. Connectivity in the VLM is like represent the ventral respiratory group, which includes the rhythm generating structures nucleus ambiguus, pre‐Bötzinger complex, and retrotrapezoid nucleus [Smith et al., 2009]. In addition the rostral VLM also contains the C1 group of adrenaline‐synthesizing neurons, which acts as a key blood pressure regulatory center [Guyenet, 2006].

In contrast, vlPAG activation elicits passive strategies, involving long duration, opioid‐dependent analgesia associated with cardiovascular and respiratory depression [Bandler et al., 2000; Keay and Bandler, 2001; Lumb, 2004]. Again our findings support these functional observations. Within the midbrain the vlPAG demonstrates connectivity to the VTA and DRN. The VTA represents part of the mesolimbic reward circuitry [Oades and Halliday, 1987] and has a role in pain and aversive processing in humans [Dunckley et al., 2005]. Moreover, the serotonergic systems within the DRN are involved in the modulation of ongoing anxiety‐related behavior and in behavioral sensitization [Abrams et al., 2004]. Connectivity in the dorsomedial medulla is likely to represent the nucleus tractus solitarius, a major relay of homeostatic information from the respiratory, cardiovascular and gastrointestinal systems [Bailey et al., 2007]. These structures have also been shown in nonhumans tracer studies to possess connections to the vlPAG [Herbert and Saper, 1992; Kirouac et al., 2004].

### Interpretation of Connectivity Patterns

Although our results demonstrate consistency in the organization of hindbrain and midbrain connections with nonhuman tracer studies, there are significant differences in the connectivity patterns to the forebrain. Connectivity between the PAG and the hypothalamus, amygdala, and PFC are derived predominantly from the vlPAG rather than the dlPAG. These structures are key to processing fear, stress and anxiety [Shin and Liberzon, 2010], which influence the homeostatic neurobiological roles of the PAG. This suggests that the vlPAG maybe involved in these processes and would certainly tie in with the hindbrain connectivity seen, relating to anxiety and aversion processing. Interestingly, these findings correlate with human fMRI studies of visceral and somatic pain processing [Dunckley et al., 2005], where correlation between PAG activation and anxiety was observed during visceral pain (vlPAG processed) but not during somatic pain (dlPAG processes).

While significant correlation between humans and nonhuman primates’ white matter anatomy has been observed using diffusion MRI [Jbabdi et al., 2013], there are some major differences, particularly within the PFC [Thiebaut de Schotten et al., 2012]. This is perhaps unsurprising given the degree of evolutionary expansion in this region [Semendeferi et al., 2001] and supports our findings of interspecies differences. It is important to remember that tractography does not inform us about precise nature of the synaptic connections made between two areas or their functional significance. These findings are important as a complement to the interpretation of functional imaging studies, particularly as high field strength fMRI becomes able to resolve activation within individual columns [Faull et al., 2015].

### Limitations

As we have alluded to in the discussion, our study does not aim to identify the number of columns in the human PAG. We have sought to identify the structure and connectivity patterns of the PAG with the prior assumption that there are four columns either side of the cerebral aqueduct [Dampney et al., 2013]. We have chosen this approach for a number of reasons. First, there is convincing evidence from cross species nonhuman experiments that the PAG is comprised of four columns and importantly these divisions are not solely based on connectivity patterns but also functional and biochemical differences [Dampney et al., 2013]. Second, we believe that inferential statistics performed to identify the optimum number of clusters, would be of limited use. This would reflect only the mathematical distinctions within the connectivity data and would not take into account the unmeasured functional and biochemical difference. Division based on identifying the most distinct clusters would likely produce two clusters of the ventral and dorsal aspects of the PAG, where connectivity differences are greatest. We believe that the resolution of a structure similar to that of the animal model of PAG when aiming to resolve four columns, demonstrates that our data fits this model of the PAG. We accept the limitations of this design but feel that the assumptions we have made are not without strong scientific merit.

### Conclusion

We have demonstrated, for the first time, subdivisions of the human PAG, as predicted by animal models. This has enabled resolution of individual column connectivity and the comparisons with animal tracer studies. Patterns of forebrain connectivity appear to be different to those found in nonhuman studies, whereas midbrain and hindbrain connectivity appears to be maintained. This suggests altered fear and anxiety processing in humans compared to animals. This study will aid the interpretation of future research into the PAG as well as translating clinically into improved planning of stereotactic interventions such as DBS.
